# Cross‐continental comparison of parasite communities in a wide‐ranging carnivore suggests associations with prey diversity and host density

**DOI:** 10.1002/ece3.7837

**Published:** 2021-07-13

**Authors:** Astrid V. Stronen, Barbara Molnar, Paolo Ciucci, Chris T. Darimont, Lorenza Grottoli, Paul C. Paquet, Tim Sallows, Judit E. G. Smits, Heather M. Bryan

**Affiliations:** ^1^ Department of Biology Biotechnical Faculty University of Ljubljana Ljubljana Slovenia; ^2^ Department of Biotechnology and Life Sciences Insubria University Varese Italy; ^3^ Department of Chemistry and Bioscience Aalborg University Aalborg Denmark; ^4^ Institute of Biology University of Neuchâtel Neuchâtel Switzerland; ^5^ Department of Biology and Biotechnologies University of Rome “La Sapienza” Rome Italy; ^6^ Department of Geography University of Victoria Victoria BC Canada; ^7^ Raincoast Conservation Foundation Denny Island BC Canada; ^8^ Hakai Institute Heriot Bay BC Canada; ^9^ Riding Mountain National Park Wasagaming MB Canada; ^10^ Department of Ecosystem and Public Health University of Calgary Calgary AB Canada

**Keywords:** *Canis lupus*, dietary diversity, direct life cycle, indirect life cycle, noninvasive monitoring, population density

## Abstract

1. Parasites are integral to ecosystem functioning yet often overlooked. Improved understanding of host–parasite associations is important, particularly for wide‐ranging species for which host range shifts and climate change could alter host–parasite interactions and their effects on ecosystem function.
2. Among the most widely distributed mammals with diverse diets, gray wolves (*Canis lupus*) host parasites that are transmitted among canids and via prey species. Wolf–parasite associations may therefore influence the population dynamics and ecological functions of both wolves and their prey. Our goal was to identify large‐scale processes that shape host–parasite interactions across populations, with the wolf as a model organism.
3. By compiling data from various studies, we examined the fecal prevalence of gastrointestinal parasites in six wolf populations from two continents in relation to wolf density, diet diversity, and other ecological conditions.
4. As expected, we found that the fecal prevalence of parasites transmitted directly to wolves via contact with other canids or their excreta was positively associated with wolf density. Contrary to our expectations, the fecal prevalence of parasites transmitted via prey was negatively associated with prey diversity. We also found that parasite communities reflected landscape characteristics and specific prey items available to wolves.
5. Several parasite taxa identified in this study, including hookworms and coccidian protozoans, can cause morbidity and mortality in canids, especially in pups, or in combination with other stressors. The density–prevalence relationship for parasites with simple life cycles may reflect a regulatory role of gastrointestinal parasites on wolf populations. Our result that fecal prevalence of parasites was lower in wolves with more diverse diets could provide insight into the mechanisms by which biodiversity may regulate disease. A diverse suite of predator–prey interactions could regulate the effects of parasitism on prey populations and mitigate the transmission of infectious agents, including zoonoses, spread via trophic interactions.

## Graphical abstract

Parasites are central to ecosystems but often overlooked. We investigated broad‐scale interactions between gastrointestinal parasites and their hosts by sampling the widely distributed gray wolf (*Canis lupus*) across six populations on two continents. As expected, wolf density was positively associated with the prevalence of fecal parasites transmitted directly between hosts. Contrary to our expectations, wolves with more diverse diets had a lower prevalence of fecal parasites transmitted via prey, an association that may reflect one mechanism by which biodiversity could influence infection patterns in hosts.

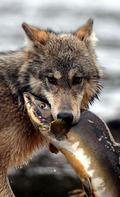

## 1 INTRODUCTION

Parasite–host relationships affect ecological processes in profound ways. As a key example, parasites can regulate host abundance by reducing host survival and fitness (Arneberg et al., 1998; Lafferty et al., 2008; Otranto, Cantacessi, Dantas‐Torres, et al., 2015; Otranto, Cantacessi, Pfeffer, et al., 2015). Parasites that exploit trophic interactions can also affect host abundance via influences on predator–prey dynamics, for example, by affecting foraging decisions by consumers (Hutchings et al., 2006) or by making prey more susceptible to predation (Lafferty et al., 2008; Lefèvre et al., 2009). Despite these important influences, parasite–host relationships are often overlooked in ecological studies (Frainer et al., 2018; Wood & Johnson, 2015).

Understanding the ecological role of parasites is essential given today's extreme levels of environmental change. For example, environmental stressors can disrupt natural balances between parasites and hosts, leading to altered transmission dynamics and host immune function (Acevedo‐Whitehouse & Duffus, 2009; Brearley et al., 2013). Biodiversity loss can also influence parasite–host relationships, for example, through the removal of nonhost species that minimize parasite transmission via dilution (Civitello et al., 2015). Moreover, the loss of parasite diversity from ecosystems may have substantial effects on ecosystem processes (Wood & Johnson, 2015). Collectively, the diverse effects of parasite–host interactions on ecosystem function combined with ongoing change call for further understanding of parasite–host relationships.

Host geographical range, population density, and body size are recognized as general predictors of parasite richness across a broad range of taxa (Kamiya et al., 2014). Parasite communities of gray wolves (*Canis lupus*) thus present a valuable system for understanding the role of parasites in host regulation and predator–prey dynamics. As top predators with few natural (i.e., nonhuman) predators, infectious disease may play a role in top‐down regulation of wolf populations, particularly by causing mortality in pups (Kreeger, 2003; Mech et al., 2008; Mech & Kurtz, 1999; Peterson et al., 1998; Seguel & Gottdenker, 2017). Parasites might also affect host vulnerability to predation (Fenton & Rands, 2006). Such infections include *Echinococcus granulosus* in moose (*Alces alces*) (Joly & Messier, 2004), which could influence broader predator–prey relationships by altering the proportion of moose versus alternate prey species in wolf diet. Moreover, wolves occupy one of the broadest ranges among terrestrial mammals, including maritime, plains, and montane regions (Mech & Boitani, 2003), with dietary differences among regional populations (Newsome et al., 2016). This diversity of habitats and diets presents an opportunity to examine general processes (e.g., host density and prey diversity) that influence the prevalence of fecal parasites across populations and differences related to specific geographical regions and habitats.

Ongoing studies of wolf–parasite associations are particularly important as emerging findings from Europe and North America have revealed new infections and spatial changes in parasitism of wolves (Beck et al., 2017; Ćirović et al., 2015; Hermosilla et al., 2017; Molnar et al., 2015, 2019; Pavlović et al., 2018) as well as in other canids (Fuehrer et al., 2016). Given that canids host several zoonotic parasites, changing host–parasite relationships could have major implications for both ecosystem and public health (Cerda et al., 2018; Tasić‐Otašević et al., 2016). Moreover, these changes combined with the long‐distance dispersal capacity of wolves and other canids also highlight the importance of establishing an early‐warning system (i.e., baseline data and ongoing monitoring programs) to identify the emergence and spread of new pathogens (Fuehrer et al., 2016).

Examining patterns in wolf–parasite associations is problematic because study designs and methods used in parasitological research often differ among studies, complicating the potential for inference from multiple studies. Despite these challenges, recent efforts have been made to compare findings across various studies to highlight wide‐scale patterns of parasite diversity (e.g., Pappalardo et al., 2020). Our aim with this comparative study, which includes different ecosystems, sampling periods, and parasitological methods, was to investigate associations between predator–prey relationships and parasite communities. Here, we combined results from three studies (Bryan et al., 2012; Molnar et al., 2019; Stronen et al., 2011) and compiled associated data on population metrics, such as wolf density and dietary diversity (Tables 1 and 2). The three studies include data from six wolf populations on two continents. All three studies employed a similar, noninvasive research approach using fecal samples to identify gastrointestinal parasites over multiple years and seasons.

**TABLE 1:** Overview of study areas for comparison of wolf gastrointestinal parasites, including number of fecal samples collected (*n*)

Acronym	Study area	Continent (country)	Habitat type	*n*	Seasons sampled^1^	Parasite detection method	Diet diversity^2^	Wolf density^3^ (wolves/1,000 km^2^)	Alternate definitive hosts^4^	History of wolf occupancy	Human disturbance^5^	References
PNALM	National Park of Abruzzo, Lazio and Molise	Europe (Italy)	Continental (montane)	88	W, P	Sodium acetate–acetic acid–formaldehyde	High (1.78)	High (50^a^)	2 (fox, dog^IV^)	Long‐term presence	HBGFL	Molnar et al. (2015)
MNP	Mercantour National Park	Europe (France)	Continental (montane)	68	W, P	Sodium acetate–acetic acid–formaldehyde	High (1.71)	Low (11.5^a^)	2 (fox, dog^III^)	Recent (recolonized 1990s)	HBGFL	Molnar et al. (2015)
YNP	Yellowstone National Park	North America (US)	Continental (montane)	186	W, P	Sodium acetate–acetic acid–formaldehyde	Low (0.19)	High (50^a^)	3 (fox, dog^I^, coyote)	Recent (re‐introduced 1990s)	B	Molnar et al. (2015)
GBR	Great Bear Rainforest	North America (Canada)	Coastal (temperate rainforest)	1556	P, S, F	Sugar flotation	Medium (0.76)	Medium (15−35^b^)	0 (dog^II^)	Long‐term presence	HTF	Bryan et al. (2012)
RMNP	Riding Mountain National Park	North America (Canada)	Continental (aspen parkland/boreal forest transition)	479	W, P, S, F	Sugar flotation	Medium (0.92)	Medium (25^c^)	3 (fox, dog^III^, coyote)	Long‐term presence	B	Sallows (2007) and Stronen et al. (2011)
DMPPF	Duck Mountain Provincial Park and Forest	North America (Canada)	Continental (aspen parkland/boreal forest transition)	122	W, P	Sugar flotation	Medium (0.92)	Medium (25^c^)	3 (fox, dog^III^, coyote)	Long‐term presence	HTBGF	Stronen et al. (2011)

Note

The six areas comprise five different habitat types, where the last two entries represent two areas from similar habitat but with different degrees of protection and human use.

^1^ W—winter (1 October–31 December), P—spring (1 January–31 March), S—summer (1 April–30 June), and F—fall (1 July–31 September). PNALM was sampled in 2006 and 2008, MNP in 2005 and 2007, YNP in 2007 and 2009, GBR in 2005–2008, RMNP in 2001–2005, and DMPPF in 2003–2005.

^2^ Classified according to the Shannon index (*H*) (Table 3).

^3^ Wolf density: (a) Molnar et al. (2015); (b) Darimont and Paquet (2000); and (c) Stronen et al. (2012). DMPPF density is unknown but believed to be like RMNP.

^4^ Dog categories: dog^I^—dogs kept on leash; dog^II^—free‐roaming and working dogs from few local communities; dog^III^—pet dogs, dogs traveling with tourists (can be seasonal), and working dogs; and dog^IV^—pet, working, stray, and feral dogs. Working dogs are shepherd dogs, livestock‐guarding dogs, hunting dogs, and bear dogs.

^5^ Activities: Hunting (H), Trapping (T), Backcountry tourism (B), Garbage including gut piles from hunting (G), Forestry (F), and Livestock (L). This table considers the territories of the investigated wolf packs, which at times extended outside protected areas.

**TABLE 2:** Percent of prey items consumed by wolves in the six study areas

Taxon	PNALM^a^	MNP^b^	YNP^c^	GBR^d^	RMNP and DMPPF^e^
Fish (Salmonidae)	—	—	—	3.4	—
Mammalia					
Carnivora	`				
Ursidae					
Black bear (*Ursus americanus*)	—	—	—	2.6	—
Phocidae					
Harbor seal (*Phoca vitulina*)	—	—	—	6.6	—
Otariidae					
California sea lion (*Zalophus californianus*)	—	—	—	1.6	—
Mustelidae					
River otter (*Lontra canadensis*)	—	—	—	2.2	—
Marten (*Martes americana*)	—	—	—	0.4	—
Perissodactyla					
Equidae					
Horses (*Equus caballus*)	14.7	—	—	—	—
Artiodactyla					
Suidae					
Wild boar (*Sus scrofa*)	28.7	0.8	—	—	—
Cervidae					
Elk/red deer (*Cervus elaphus*)	7.1	13.4	96	—	67.1
Mule deer (*Odocoileus hemionus*)	—	—	1.5	82.6	—
White‐tailed deer (*Odocoileus virginianus*)	—	—	—	—	4.0
Moose (*Alces alces*)	—	—	1.5	—	22.9
Roe deer (*Capreolus capreolus*)	11.2	2.9	—	—	—
Bovidae					
Cattle (*Bos taurus*)	17.7	—	—	—	—
Bison (*Bison bison*)	—	—	0.5	—	—
Domestic sheep (*Ovis aries*) and goat (*Capra aegagrus hircus*)	18.2	14.6	—	—	—
Mountain goat (*Oreamnos americanus*)	—	—	—	0.1	—
Ibex (*Capra ibex*)	—	22.9	—	—	—
Chamois (*Rupicapra rupicapra*)	2.4	32.6	—	—	—
European Mouflon (*Ovis aries musimon*)	—	8.6	—	—	—
Rodentia					
Castoridae					
Beaver (*Castor canadensis*)	—	—	—	0.4	5.3
Unknown rodent	—	—	—	0.1	—
Lagomorpha					
Leporidae					
Snowshoe hare (*Lepus americanus*)	—	—	—	—	0.7
Unknown Mammal	—	2.8	—	—	—

^a^ Mammalian prey biomass from 660 fecal samples collected during the winter and spring 2006–2008 (Grottoli, 2011).

^b^ Mammalian prey biomass from 155 fecal samples in winter and spring 1997–2001 (Espuno, 2004).

^c^ Observations of 211 wolf kills in winter and spring 1995–2000 in the Northern Range of YNP (Smith et al., 2004).

^d^ Derived from biomass estimates of mammalian prey in 2,203 fecal samples in spring, summer, and fall 2001–2003 (Darimont et al., 2008). Estimate of salmon consumption based on isotopic analysis of wolf hair (Semmens et al., 2009). All biomass estimates were then scaled by the new total including salmon (103.5%) to sum to 100%.

^e^ Mammalian prey biomass from 369 feces collected between fall 2001 and summer 2003 (Sallows, 2007).

Fecal samples have the advantage of being noninvasive and relatively inexpensive to collect; however, parasite detection and quantification is complicated by temporal variability in the shedding of larval stages (i.e., the eggs, oocysts, sporocysts, cysts, and larvae of helminths and protozoans) in feces (Branda et al., 2006; Cartwright, 1999). We therefore focused our comparative analysis on differences in fecal prevalence (i.e., proportion of fecal samples in which a parasite's larval stage was detected) rather than parasite richness and intensity, which are particularly sensitive to detection and quantification. Notably, fecal prevalence of larval stages represents the level of environmental contamination and potential for transmission of common parasites among hosts, including wolves and prey species that serve as intermediate hosts. Our overall goal was to identify processes that shape the prevalence of fecal parasites across populations over broad spatial and temporal scales. Seasonal differences often affect wolf diet and parasite prevalence, which have been found to vary among prey species, parasite species, packs, and years (Bryan et al., 2012; Ciucci et al., 2018, 2020; Sallows, 2007). Our specific objectives were therefore to examine whether fecal prevalence of parasites was associated with (a) wolf density, which could indicate density‐dependent transmission and a potential regulatory role of parasites on wolves; (b) dietary diversity, which could influence the parasite taxa to which wolves are exposed via trophic interactions; or (c) study areas, which represent a proxy of environmental variables that influence wolf–parasite associations. In addition, we examined potential associations of parasitic prevalence with the number of alternative hosts and the recent history of each wolf population (i.e., historical presence vs. recolonization).

To achieve our first two objectives, we classified parasites into broad categories according to life cycle. Specifically, parasites with direct life cycles are those that require only one host to complete their life cycles (i.e., are transmitted directly among wolves or other suitable hosts). For parasites with direct life cycles, higher wolf densities might facilitate transmission; therefore, we predicted that the fecal prevalence of parasites with direct life cycles would be positively correlated with wolf density. Alternatively, parasites with direct life cycles could be transmitted to wolves via other canids, including domestic dogs (*C. l*. *familiaris*). Accordingly, if alternative hosts are primarily responsible for transmission, the fecal prevalence of parasites with direct life cycles might be correlated more strongly with the diversity or abundance of alternative host species than with wolf density. Parasites with indirect life cycles are those that have a larval stage that requires one or more intermediate hosts to complete its life cycle (i.e., are transmitted to wolves via prey). For parasites with indirect life cycles, the relationship between diet diversity and the prevalence of fecal parasites could be even more complex. On one hand, given that wolves are exposed to parasites from many prey species, and that parasite intensity may be limited within a host by competition for resources (e.g., attachment sites in the gastrointestinal tract), the fecal prevalence might be similar across study areas regardless of variation in wolf diet. In this case, we would expect no relationship between the fecal prevalence of parasites with indirect life cycles and dietary diversity. On the other hand, parasitic diversity often correlates with host diversity (Lafferty et al., 2008), so ecosystems with a higher diversity of either definitive hosts, including domestic or wild carnivores, or intermediate hosts (i.e., prey species for definitive hosts) might have higher parasitic diversity. To test these competing hypotheses for parasites with direct and indirect life cycles, we assessed the effect of diet diversity, wolf density, and number of alternative hosts on fecal parasite prevalence. We also included recent history of wolf residency as a predictor in our models, as parasite communities may differ among established and recolonizing wolf populations (Molnar et al., 2019). To address our third objective of examining differences among study areas, we compared parasitic larval stages identified across study areas and interpreted our findings in relation to the life history and ecological requirements of specific parasite groups.

## 2 MATERIALS AND METHODS

### 2.1 Study areas and sample collection

**FIGURE 1:**
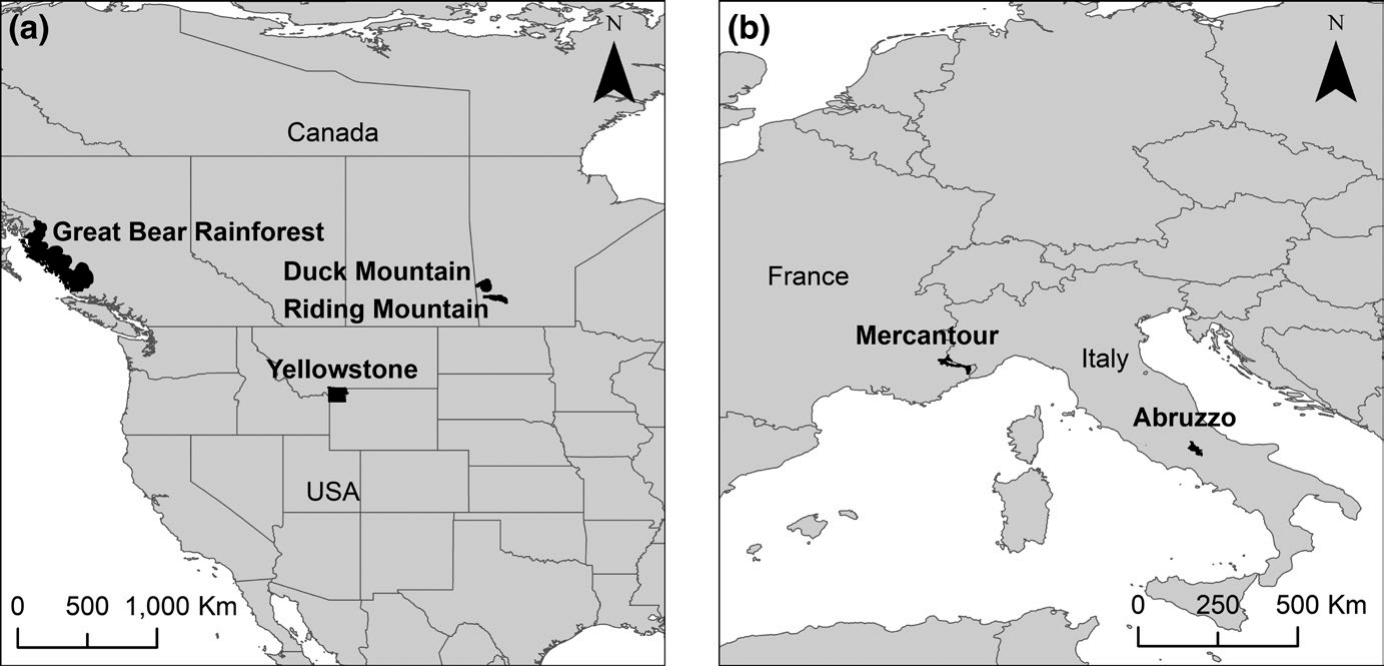
Wolf fecal samples were collected from six study areas, including the Great Bear Rainforest (GBR), Duck Mountain Provincial Park and Forest (DMPPF), Riding Mountain National Park (RMNP), and Yellowstone National Park (YNP) in North America (a), and Mercantour National Park (MNP) and National Park of Abruzzo, Lazio, and Molise (PNALM) in Europe (b). Park boundaries downloaded from www.protectedplanet.net or http://mli2.gov.mb.ca/adminbnd/index.html and country maps from http://www.naturalearthdata.com on 10 July 2018

Our study areas included two in Europe, one in the United States, and three in Canada (Table 1; Figure 1a,b). In all study areas, sampling occurred via noninvasive collection of fecal samples from roads, trails, kill sites, and other areas used by wolves. In Europe, samples were collected from Abruzzo, Lazio and Molise National Park (PNALM) in Italy (*n* = 88) and Mercantour National Park (MNP) in France (*n* = 68) (Molnar et al., 2015, 2019). Samples from the United States were collected in the Northern Range of Yellowstone National Park (YNP) (*n* = 186) (Molnar et al., 2015, 2019). In Canada, samples were collected from the central coast of British Columbia in a region known as the Great Bear Rainforest (GBR) (*n* = 1,556) (Bryan et al., 2012), and from Riding Mountain National Park (RMNP) (*n* = 479) and Duck Mountain Provincial Park and Forest (DMPPF) (*n* = 122) in Manitoba, in the transition zone between the Prairie and Boreal Plain ecozones (henceforth Prairie–Boreal region) (Ehlrich et al., 1956; Love, 1959; Sallows, 2007; Stronen et al., 2011). Our combined dataset includes data from three independent studies and six sampling areas that vary in ecological characteristics (Table 1) and wolf diet (Table 2). Within each study area, samples were collected from a broad spatial scale (ranging from 5,000 to 60,000 km^2^) and from multiple wolf social groups. Sampling at each site spanned at least two seasons and occurred over 2–4 different years. Due to differences in the timing and spatial extent of sampling among the six studies, we were unable to include the effects of year and season in our analysis. Moreover, we did not have comparably detailed information on social groups or individual wolf identity in all study areas. Consequently, we pooled parasite data collected from all feces within each study area. Although this limits our ability to draw inferences about spatial and temporal variability in wolf–parasite associations, our analysis nonetheless provides a coarse‐scale overview of fecal parasite prevalence across the six study areas (Table 2).

Samples from Bryan et al. (2012) were collected under the University of Saskatchewan's Animal Care Committee Protocol 2007009 and with permission of the Heiltsuk, Kitasoo/Xai'xais, Gitga'at, and Wuikinuxv Nations. Samples in RMNP were collected under Environmental Assessment permit numbers RMNP000396 and RMNP000477, and permission was obtained from the Manitoba provincial government to collect fecal samples in the Duck Mountain Provincial Park and Forest. Research in PNALM was approved by the national Park Authority (Determination No. 38 dated 24 March 2003), whereas MNP did not require any specific permission for collection of fecal samples. In YNP, fieldwork and processing of samples was conducted under permit numbers YELL‐2007‐SCI 5716, YELL‐2008‐SCI 5716, and YELL‐2009‐SCI 5716.

### 2.2 Identification of larval stages of parasites

Samples from all study areas were frozen at −20°C before analysis. Larval stages (i.e., eggs, oocysts, sporocysts, and nematode larvae) of the parasites in wolf feces collected from the three Canadian study areas (RMNP, DMPPF, and GBR) were identified at the University of Saskatchewan using a modified Wisconsin sugar flotation method on 4 g of feces followed by microscopy (Foreyt, 1989, 2001; Stronen et al., 2011). For YNP, MNP, and PNALM, helminth eggs and protozoan cysts were identified using a sodium acetate–acetic acid–formaldehyde (SAF) procedure on 1.55 g of feces followed by microscopy (Molnar et al., 2015). Both flotation and SAF methods are well suited to identify the presence of helminth eggs and protozoan cysts in fecal samples; however, the ability of the two procedures to detect parasite stages may differ by taxa. The flotation method uses more fecal material (4 g) than the sedimentation method (1.55 g) and should therefore be more sensitive. The sedimentation method, however, may be better than flotation for detecting heavier larval stages, such as the eggs of trematodes and Taeniid cestodes, which may in part make up for the lower sensitivity related to sample volume for some parasite taxa (Öge et al., 2017; Wolf et al., 2014). We applied correction factors based on published comparisons of parasite detection methods to examine the potential effects of methodological differences on our results (Appendix S1). Results using the dataset where we corrected for methodological differences in the fecal flotation used in GBR, RMNP, and DMPFF were similar to those obtained using the original dataset (Appendix S1; Figures S1 and S2; Table S2).

Microscopy procedures were similar across studies; for each sample, the entire slide was examined under ×100 total magnification with spot checks at ×400 magnification. Parasite stages were identified to the lowest possible taxonomic level. In some cases, identifying species was possible; however, parasite stages within a genus often are difficult to differentiate at the egg/oocyst stage and thus were reported only to genus or to family in the case of Taeniid cestodes. For the three Canadian study areas, feces were also examined for the presence of the protozoans *Giardia* spp. and *Cryptosporidium* spp., which were identified by immunofluorescence using the Cyst‐a‐GloTM Comprehensive Kit (Waterborne Inc.) with modifications described in Stronen et al. (2011).

### 2.3 Data preparation and analysis

To focus on helminth and protozoan endoparasites of wolves, the ectoparasite *Demodex* spp. and endoparasites *Moniezia* and *Soboliphyme* spp.—whose definitive hosts are thought to originate in the prey consumed by the wolves rather than in wolves themselves—were excluded from the comparison. Unidentified nematode larvae—which may have represented free‐living nematodes or nematode parasites of prey—were also excluded due to potential differences in detection and identification among studies combined with a low fecal prevalence (Bryan et al., 2010, 2012; Molnar et al., 2019). For taxa that had been identified to species or genus in one study, but to genus or order in another, the higher‐level classification was used. Specifically, parasites classified as *Isospora* by Bryan et al. (2012) and Molnar et al. (2015) were classified as Coccidea to be consistent with Stronen et al. (2011). Similarly, *Capillaria aerophila* and *C. boehmi*, which were classified separately by Molnar et al. (2019), were grouped under the genus *Capillaria* to be consistent with the other two studies. Parasites of unknown identity were also left out of the comparative analysis.

Parasitic taxa retained for the comparison were classified based on their life cycles as having either direct transmission or indirect transmission, the latter requiring one or more intermediate hosts and being transmitted to wolves via consumption of prey. Data on the waterborne parasite, *Giardia*, were only available from the GBR, RMNP, and DMPPF study areas and were therefore analyzed separately. Characteristics of each study area, including wolf density, dietary diversity, wolf population history, number of alternative hosts, and level of human disturbance, were obtained from published literature and local knowledge of each study area (Tables 1, 2, 3). The seasonal timing of wolf dietary data matched the timing of parasite data collected within study areas, but collection years differed in some study areas (e.g., RMNP dietary data were collected 2001–2003; parasitic data 2001–2005). The dietary data compiled for each study area, including proportions of each prey item (p_i_; Table 2) and the number of prey species consumed in each area (*S*; Table 3), were used to calculate dietary diversity using Shannon's diversity (*H*) and equitability indices (*E_H_*). These indices consider the number and relative proportions of dietary items and are linearly related, with *E_H_* being *H* scaled between 0 and 1 (Table 1).

**TABLE 3:** Diversity indices of prey items in the diet of wolves in each study area, calculated using prey species and proportions listed in Table 2

Diversity index	PNALM	MNP	YNP	GBR	RMNP and DMPPF
Number of species (*S*)	7	8	4	10	5
Shannon index (*H*)	1.78	1.71	0.19	0.76	0.92
Equitability or evenness (*E_H_*)	0.91	0.82	0.14	0.33	0.57

We compared the fecal prevalence (the proportion of feces with at least one parasite) among study areas graphically in relation to wolf density and dietary diversity. To support this qualitative analysis, we used binomial generalized linear mixed effects models with a logit link function to assess whether wolf density, dietary diversity, wolf population history, or number of alternative hosts affected the fecal prevalence of parasites with direct and indirect life cycles. To account for differences among study areas, we included study area as a random effect. We included only one predictor per candidate model because we had only one measure of each variable for each of the six sites (i.e., an effective sample size of six). Models were ranked using Akaike information criterion corrected for small sample size (AIC_c_). We found that AIC scores were lower or similar when independent variables were included as continuous or categorical predictors; therefore, we included all variables except wolf population history as continuous variables in the final models. We used a simulation to examine potential effects of methodological differences (i.e., fecal flotation vs. sedimentation) on our comparisons of parasites with direct and indirect life cycles among study areas (Appendix S1). Accordingly, we used published data comparing the two approaches to identify correction factors for each parasitic taxon (Table S1 in Appendix S1). We then randomly added or removed positive fecal samples depending on whether a particular taxon was over‐ or under‐reported using sedimentation compared with flotation. We then recreated figures and reran the statistical analysis to evaluate the effects of methodological differences on our results and interpretations (Figures S1, S2 and Table S2 in Appendix S1). Figures and analyses were done using R (R Development Core Team, 2017, version 3.3.3).

## 3 RESULTS

Thirteen parasitic taxa were retained for comparison among study areas. Directly transmitted taxa included the nematodes *Toxascaris leonina*, *Capillaria* spp., *Toxocara canis*, *Trichuris* spp., *Uncinaria* spp., and Spiroidea as well as the protozoan subclass Coccidea. Indirectly transmitted parasite taxa included cestodes in the Taeniid family and genus *Diphyllobothrium* spp., trematodes *Alaria* spp. and *Metorchis* spp., the nematode *Physaloptera* spp., and the protozoan *Sarcocystis* spp.

### 3.1 Parasites with direct life cycles

**FIGURE 2:**
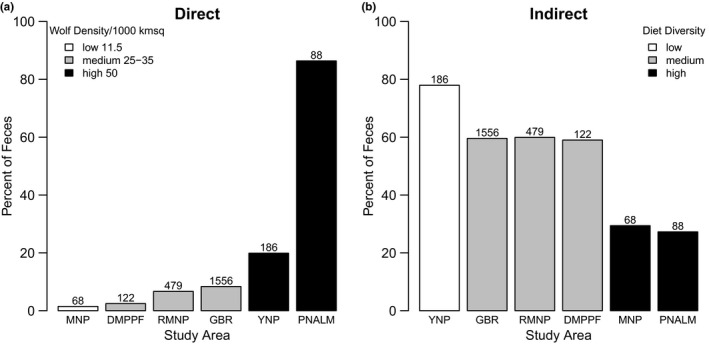
Percentage of wolf parasites with direct and indirect life cycles per study area in relation to estimated wolf density (individuals per 1,000 km^2^) and dietary diversity (see Table 2 for details). These are Abruzzo National Park (PNALM, Italy), Mercantour National Park (MNP, France), Yellowstone National Park (YNP, US), the Great Bear Rainforest (GBR) in coastal Canada, and Riding Mountain National Park (RMNP) and Duck Mountain Provincial Park and Forest (DMPPF) in continental Canada. Study areas are ordered by increasing wolf density (panel a) and diet diversity (panel b)

**FIGURE 3:**
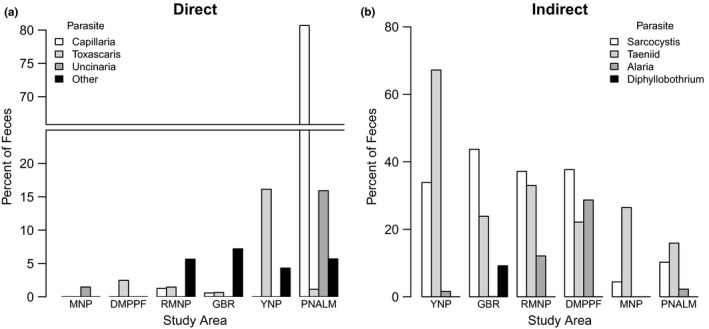
Percentage of wolf parasites with direct (a) and indirect (b) life cycles summarized for each study area. These are Abruzzo National Park (PNALM, Italy), Mercantour National Park (MNP, France), Yellowstone National Park (YNP, US), the Great Bear Rainforest (GBR) in coastal Canada, and Riding Mountain National Park (RMNP) and Duck Mountain Provincial Park and Forest (DMPPF) in continental Canada. Study areas are ordered by increasing wolf density (panel a) and dietary diversity (panel b). For panel a, “Other” includes parasite taxa *Toxocara canis*, *Trichuris* spp., Spiroidea, and Coccidia. Not shown in panel b are parasite taxa *Metorchis* spp., which occurred in 0.6% of samples from the GBR, and *Physaloptera* spp., which occurred in 2.3% of samples from PNALM

Comparison of parasites with direct life cycles in wolves showed that the highest fecal prevalence occurred in PNALM and YNP, which also had the highest wolf densities (Figures 2 and 3a). This was followed by the three areas GBR, RMNP, and DMPPF with medium wolf density. MNP, the only area with low density, exhibited the lowest fecal prevalence of parasites with direct life cycles. Comparison of models built with a single predictor variable each revealed that wolf density was the strongest predictor of the fecal prevalence of parasites with direct life cycles relative to models comprised of either dietary diversity, number of alternative hosts, or wolf population history (Table 4). The model with wolf density was the only one that differed from the null model, and revealed that the fecal prevalence of parasites with direct life cycles increased with increasing wolf density. Specifically, fecal samples were 7.1 times (95% Confidence Interval: 2.5–20.5) more likely to be positive for parasites for each increase in the level of wolf density (i.e., low, medium, and high, ranging from 11.5 to 50 wolves/km^2^). The high fecal prevalence of parasites with direct life cycles in PNALM was explained primarily by the presence of a single group, *Capillaria* spp. (80% prevalence), a parasite of the respiratory system, as well as by the hookworm *Uncinaria* spp. (15% prevalence). In contrast, YNP wolves exhibited primarily the parasite group *Toxascaris leonina* (15% prevalence), which is usually found in pups but rarely in adults.

**TABLE 4:** Candidate models for predicting the presence of parasites with direct or indirect life cycles in wolf feces, including Akaike information criterion (AICc) ranking and weight, coefficient estimates and standard error (*SE*), and 95% confidence intervals (CI)

Response	Predictor	*df*	AICc	ΔAICc	Model weight	Estimate ± *SE*	Lower, Upper 95% CI
Parasites with direct life cycles	Wolf density	3	1,451.38	0	0.88	1.96 ± 0.54	0.90, 3.02
	Null (~1)	2	1,457.10	5.72	0.05		
	Diet diversity	3	1,458.54	7.16	0.02	0.67 ± 0.85	−1.00, 2.34
	History of recolonization	3	1,458.72	7.34	0.02	−1.08 ± 1.74	−4.49, 2.33
	Alternative hosts	3	1,459.07	7.69	0.02	−0.18 ± 1.05	−2.24, 1.89
Parasites with indirect life cycles	Diet diversity	3	3,308.47	0	1	−0.82 ± 0.12	−1.06, −0.57
	Null (~1)	2	3,321.72	13.26	0		
	Wolf density	3	3,323.25	14.78	0	0.24 ± 0.34	−0.43, 0.90
	Alternative hosts	3	3,323.41	14.94	0	0.22 ± 0.40	−0.55, 1.00
	History of recolonization	3	3,323.67	15.1	0	0.17 ± 0.67	−1.15, 1.48

Note

Study area was included in all models as a random effect. Wolf density, dietary diversity, and number of alternative hosts were centered and scaled to have a mean of 0 and standard deviation of 1. History of recolonization was coded as “Recent” or “Not Recent.”

### 3.2 Parasites with indirect life cycles

Contrary to our expectations, comparison of parasites with indirect life cycles showed clear differences in fecal prevalence among areas (Figures 2 and 3b). Most notably, fecal prevalence was lower in areas with higher dietary diversity (Table 4). Comparison of models comprising a single predictor variable each supported a negative association between dietary diversity and fecal prevalence of parasites with indirect life cycles, with dietary diversity emerging as the top model, and the only model that differed from the null model. Specifically, parasites with indirect life cycles were 0.44 times less likely (95% confidence interval: 0.35–0.57) to occur in fecal samples for each level of increasing dietary diversity (i.e., with a Shannon's index [*H*] ranging from 0.92 to 1.78).

### 3.3 Parasites associated with specific study areas

Although several parasite taxa were common across study areas (e.g., Taeniids), others were study area specific as associated with different landscapes inhabited by wolves. In particular, the taxa *Diphyllobothrium* spp. and *Metorchis* spp. were linked to aquatic environments and consumption of fish and therefore were detected in GBR but not elsewhere. In addition, we observed that *Alaria* spp., a parasite also linked with aquatic environments, was common in DMPPF (ca. 30% prevalence) and RMNP (ca. 13%) wolves, where freshwater and wetlands are abundant.

### 3.4 Giardia

**FIGURE 4:**
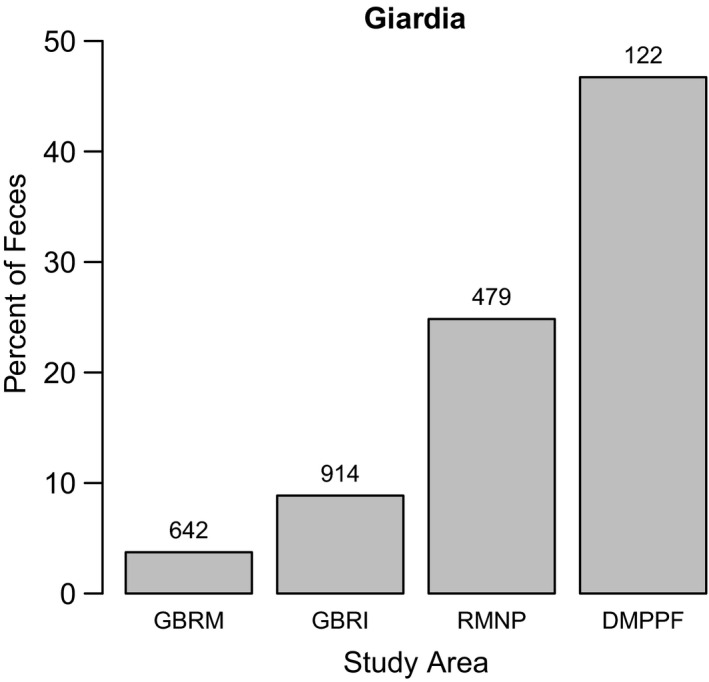
*Giardia* spp. prevalence in each study area for coastal and continental Canada. These areas comprise the Great Bear Rainforest Island (GBRI) and Mainland (GBRM) areas in coastal Canada, and Riding Mountain National Park (RMNP) and Duck Mountain Provincial Park and Forest (DMPPF) in continental Canada

The prevalence of *Giardia* spp. differed between Pacific Coastal and Prairie–Boreal habitats (Figure 4). Specifically, prevalence was 6.8% in the GBR, whereas it was 21.9% and 46.7% in RMNP and DMPPF, respectively. Samples from the GBR harbored *Giardia* assemblages A (*G. duodenalis*, *n* = 3) and B (*G. enterica*, *n* = 11) that are both zoonotic. By contrast, the samples from RMNP (*n* = 15) harbored *Giardia* assemblage B (*n* = 5), C (*n* = 6), and D (*n* = 4), where the assemblages C and D (*G. canis*) are hosted by dogs and other canid species.

## 4 DISCUSSION

Our results from wolf populations sampled in diverse ecological settings in Europe and North America suggest a relationship between host density and the fecal prevalence of parasites with direct life cycles and show that dietary diversity may affect the fecal prevalence of parasites with indirect life cycles.

### 4.1 Parasites with direct life cycles

For parasites with direct transmission, our findings most strongly support a density‐dependent relationship between fecal prevalence of parasites and wolf density across populations. A similar relationship has been demonstrated in nematodes across 19 mammalian species (Arneberg et al., 1998) and relates to increased transmission probabilities as host density increases. Pup mortality and the prevalence of canine distemper virus were both higher in an area of higher wolf density (Almberg et al., 2009), and the relationship between population density and risk of exposure was supported by a broad‐scale analysis of pathogens in North American wolves (Brandell et al., 2021). Although gastrointestinal parasites often have minimal effects on host populations, some directly transmitted parasites in this comparative study, including hookworms in the genera *Uncinaria* and *Toxocara* and Coccidian protozoans, can cause morbidity or mortality in canids (e.g., Kreeger, 2003; Mech & Kurtz, 1999; Seguel & Gottdenker, 2017), especially in pups or in combination with other stressors (Hudson et al., 2006). If mortality due to these parasites with direct life cycles increases with higher density within populations, then our findings imply that gastrointestinal parasites might play a role in regulating wolf population size.

The density–prevalence relationship may also have implications for changing densities of alternative hosts. Although the number of alternative hosts was not a strong predictor of prevalence of fecal parasites across study areas, our analysis did not consider the densities of alternative hosts. For example, PNALM had the highest density of dogs (Molnar et al., 2019), which, at least in part, may explain the high prevalence of *Capillaria* spp. in that study area. If the abundance of dogs or other alternative hosts increases relative to wolf density, then parasite prevalence might no longer be associated with wolf density. Therefore, monitoring presence and abundance of parasite species in relation to changes in canid communities could help identify changes in multihost–parasite assemblages. Recent analyses have also reported an inverse relationship between individual levels of genomic variation and infection severity of a sarcoptic mange caused by the ectoparasitic mite *Sarcoptes scabiei* (DeCandia et al., 2021), which points to the importance of maintaining genetic diversity and the long‐term risks for small populations surrounded by potential alternate host species.

Different coprological techniques used to detect parasites may have influenced our results showing high prevalence of the nematodes *Capillaria* spp. and *Uncinaria* spp. in PNALM and *Toxascaris leonina* in YNP. Specifically, SAF sedimentation was used on feces collected in PNALM, YNP, and MNP, and a modified Wisconsin flotation procedure was applied in the other three areas. A comparative analysis of gastrointestinal parasites in reptiles found that the detection of nematode eggs was 13.6% higher using flotation than sedimentation (Wolf et al., 2014). However, our simulation indicated that the methodological differences did not have any major effects on the findings for the taxa we examined. Moreover, because we found the highest prevalence of nematodes in samples analyzed with SAF sedimentation—the technique expected to produce more conservative results for nematodes—our results suggest that these nematodes were indeed more prevalent in wolf feces collected from PNALM and YNP compared with the other study areas. Such a pattern aligns with the relationship we observed between higher wolf density and higher prevalence of parasites with direct life cycles.

### 4.2 Parasites with indirect life cycles

In contrast with our original prediction, the overall fecal prevalence of parasites with indirect life cycles differed among study areas in relation to dietary diversity. Notably, the fecal prevalence of parasites with indirect life cycles was lowest in Europe, where wolves consume a wide variety of prey that includes wild and domestic species. By contrast, parasite prevalence was higher in the three North American study areas where wolves specialize on fewer species, with the highest parasite fecal prevalence occurring in YNP where elk comprise >95% of wolves' winter diet. The difference in fecal prevalence between continents was driven largely by *Sarcocystis* spp., which was lower in wolves from Europe compared with the three North American study areas. Although *Sarcocystis* spp. infects prey species consumed by wolves in Europe and North America, different species of *Sarcocystis* differ in host specificity, especially in their definitive hosts. Wolves with more diverse diets could potentially be exposed to fewer wolf‐specific *Sarcocystis* species, although this possibility warrants further research. Differences in the fecal prevalence of *Sarcosystis* spp. sporocysts and Taeniid eggs in wolf feces could also reflect different prevalence among prey species.

Differences in the fecal prevalence of parasites with indirect life cycles may have been influenced by methodological differences in coprology among studies. For example, Wolf et al. (2014) found that the flotation technique had better detection capabilities for protozoan oocysts than SAF sedimentation. Therefore, the methodology could have influenced the apparent higher prevalence of *Sarcocystis* in GBR, RMNP, and DMPPF compared with PNALM and MNP. However, the simulation results suggested that methodological differences did not have a major effect on the detection of parasites with indirect life cycles. Additionally, the prevalence of *Sarcocystis* spp. in YNP was also higher than in the European study areas PNALM and MNP, all of which used SAF sedimentation. The trematode *Alaria* spp. was most common in the North American study areas RMNP and DMPPF examined with flotation, whereas trematodes have been reported to be more detectable with SAF (Wolf et al., 2014). For the cestode *Diphyllobothrium* spp. and the Taeniid family, we have not found published records of comparative studies. However, both the highest prevalence and the lowest prevalence were detected with SAF. For *Diphyllobothrium* spp., it is possible that we have missed detection in some of our study areas, although our results are consistent with the feeding ecology of coastal wolves.

Although unexpected, a negative relationship between dietary diversity and the fecal prevalence of parasites with indirect life cycles is plausible and could occur via multiple mechanisms. For example, diverse diets could reduce wolf exposure to parasitic species that occur in any single, dominant prey species, thereby reducing overall infection rates. Exposure to different parasites from multiple prey species could also reduce overall infection rates via interspecific competition among parasites in the digestive tract of wolves or other final hosts (Mideo, 2009). Regardless of the mechanism, our results appear consistent with the findings of Friesen and Roth (2016), who suggested that wolves' use of alternate prey such as beavers (*Castor canadensis*)—which do not serve as alternate host to many wolf parasites—may limit cestode intensity in wolves and ultimately reduce parasite density in local ungulates.

Although differences in wolf density, in the number of alternative hosts, and in wolf population history among study areas did not emerge as top predictors of the fecal prevalence of parasites with indirect life cycles, these variables may have contributed to the observed results in some study areas. For example, the high fecal prevalence of Taeniid tapeworms in wolves from YNP might reflect the recent re‐introduction of wolves and associated changes in parasite–host communities (Molnar et al., 2019). Interestingly, Taeniid fecal prevalence was lower in wolves from MNP with a similarly recent history of recolonization, suggesting either that recolonization does not influence parasite fecal prevalence, that the process of recolonization by wolves and their parasites differed in these two populations (e.g., re‐introduction of dewormed wolves in YNP vs. natural recolonization of wolves with their own parasite communities in MNP), or that other differences between the two areas—such as the higher diversity of prey species in MNP—influence Taeniid fecal prevalence. Finally, numerous biological and ecological variables, including combinations of variables (e.g., high wolf density and high prey density), could also influence the fecal prevalence of parasites with indirect life cycles and could be examined in further studies.

The relationship between dietary diversity and fecal prevalence of parasites warrants further study in part because of the potential implications for the long‐term health of predator–prey relationships and ecosystem function. Although gastrointestinal parasites may have low pathogenicity in healthy, unstressed definitive hosts, the effects of parasites may differ when combined with stressors such as food shortages or human disturbance, including contaminants (Marcogliese & Pietrock, 2011). Therefore, minimizing parasite burden by ensuring wolves have access to alternative prey may be beneficial to wolves. In addition to affecting wolf health, lower prevalence of parasites in wolves with higher diet diversity may reduce parasite prevalence in prey species by decreasing the number of parasite larval stages spread via wolf feces (Joly & Messier, 2004).

### 4.3 Environmental influences on the distribution of specific parasitic taxa

Our study revealed differences related to specific prey items for which access differs sharply among the six study areas. The most striking example was that samples from coastal marine habitats yielded *Diphyllobothrium* spp. and *Metorchis* spp., known to be associated with the consumption of marine resources (Bryan et al., 2012). *Diphyllobothrium* spp. has also been reported in wolves from the Canadian Northwest Territories (Choquette et al., 1973; Schurer et al., 2016) and from Croatia (Hermosilla et al., 2017) where wolves presumably had access to marine or freshwater fish. Moreover, there are reports of wolves catching and consuming freshwater fish in continental areas far from the ocean. These include the Northwest Territories were Kuyt (1969) noted the use of lake trout (*Salvelinus namaycush*), northern pike (*Esox lucius*) and potentially other species as important for wolf diet in areas without access to caribou (*Rangifer tarandus*) during the denning season, and in Minnesota where wolves were recently reported to have consumed northern pike and possibly other fish species (Gable et al., 2018). This association between wolves and aquatic diets seems ancient (Meachen et al., 2020). Although it is unknown whether such behavior is common and shared with other wolves in the region, wolves can learn to hunt species to which they have never been exposed previously (Smith et al., 2000).

Detecting rare prey items in wolves, however, can be challenging, especially as dietary analysis of wolf feces typically focuses on mammalian prey items. As a key example, *Alaria* spp. was detected in feces from wolves in the Prairie–Boreal region and in Italy (PNALM). *Alaria* typically uses species such as frogs and tadpoles (Anura), and snakes (Serpentes) as intermediate hosts, and might therefore represent ingestion of these prey items by wolves. In Europe, however, *Alaria alata* has been identified in tissues of wild boar, representing a potential source of infection for wolves (Bagrade et al., 2009; Strokowska et al., 2020). Although wild boar comprised >30% of wolf diet in PNALM, the relatively low fecal prevalence of *Alaria* spp. could occur if the prevalence in boars was also low at the time of sampling. Conceivably, mammalian prey in Canada could also serve as intermediate hosts for *Alaria* spp. transmission to wolves. Parasitic larval stages, which may be present in feces even when the original prey item is not present, may therefore be useful for detecting novel, seasonal, or rare prey items used by wolves or for identifying novel parasite–host associations.

### 4.4 Giardia

We also found differences in the fecal prevalence and composition of *Giardia* spp. between the Coastal and Prairie study areas. Interestingly, prevalence of *Giardia* spp. was three and six times higher in RMNP and DMPPF, respectively, compared with the GBR. Within the GBR, *Giardia* spp. prevalence was higher in samples collected from islands compared with those from the mainland (Bryan et al., 2012). Collectively, these findings suggest that landscape characteristics, such as the abundance of and access to freshwater, could play an important role in the distribution and prevalence of *Giardia* spp. in wolves, the frequency of transmission being facilitated by water (Thompson & Ash, 2016). Although the number of genotyped samples from each study area was modest, we found interesting differences in the composition of *Giardia* assemblages. Assemblages with zoonotic potential (A and/or B; reviewed in Thompson & Ash, 2016) were detected in both Pacific Coastal and Prairie regions, appearing to be broadly distributed in wolves and other wildlife in western Canada (Schurer et al., 2016; Thompson & Ash, 2016). By contrast, assemblages thought to be specific to canids (C and D) were only detected in samples from the Prairies (Thompson & Ash, 2016). Future surveys could investigate possible signs of change in *Giardia* prevalence or assemblage composition, such as geographical expansion of canid‐specific assemblages. *Giardia* infection might be a particular concern for transmission between wild and domestic species, which could have important zoonotic potential even at low prevalence, especially where wolf territories bring them into close contact with domestic animals and humans (Hermosilla et al., 2017).

### 4.5 Limitations of fecal‐based, comparative studies of diet and parasitism

#### 4.5.1 Diet and season

Importantly, seasonal differences often affect wolf diet and parasite prevalence, which have been found to vary among prey species, parasite species, packs, and year also in our study areas (Bryan et al., 2012; Ciucci et al., 2020; Sallows, 2007). In the Prairie–Boreal region, Sallows (2007) found elk to be the most common wolf prey species in all seasons, whereas beaver, moose, and white‐tailed deer (*Odocoileus virginianus*) showed seasonal variation. Unlike diet, parasite communities in wolf feces might not be expected to change seasonally as many parasite taxa can survive in their hosts for months to years, potentially transcending seasons. Nonetheless, in Pacific Coastal wolves, *Diphyllobothrium* spp. eggs were more than seven times more likely to occur in feces collected in autumn relative to feces collected during spring in Pacific Coastal wolves, whereas Taeniid eggs and *Sarcocystis* spp. sporocysts were 0.31 and 0.48 times, respectively, less likely to occur in feces collected in autumn compared with those collected in spring (Bryan et al., 2012). These seasonal changes were linked with the consumption of salmon (*Onchorynchus* sp.), the intermediate host for *Diphyllobothrium* spp., in autumn and a correspondingly lower consumption of Sitka black‐tailed deer (*O*. *hemionus sitkensis*), the most likely intermediate host for Taeniid tapeworms and *Sarcocystis* spp. This seasonal variation in both diet and fecal parasite prevalence supports our approach of matching the seasons in which dietary and parasite data were collected. Whereas some prey species and parasites are therefore likely to be influenced by sampling seasons—sometimes in a known manner and often not—analyses of parasite prevalence combined with regional knowledge of available prey species and wolf diets can nonetheless offer insights across study areas about broader trends and questions for further monitoring and research in rapidly changing environments.

#### 4.5.2 Coprological methods

Obtaining consistent results on parasite presence given irregular excretion of parasites can be difficult (Branda et al., 2006; Cartwright, 1999), even in heavily infected individuals (Marti & Koella, 1993). Moreover, direct comparison examining the same fecal samples with flotation and SAF methods has demonstrated that there can be significant differences in detection, where some taxa are better detected by SAF and others by flotation (Wolf et al., 2014). We were unable to perform direct comparison of the original samples from our earlier studies, and some parasite taxa may have been missed or under‐reported because of methodological differences or because of sampling was done during a time when there was less shedding of parasite eggs. Instead, we used a simulation approach to adjust for potential biased introduced by methodological differences among study areas. Although the simulation was based on published literature whose methods, host species, and parasite taxa differed somewhat from ours (Appendix S1), the results suggested that our main findings would not be greatly affected by methodological differences and could be useful in future monitoring and research.

## 5 CONCLUSIONS

Tracking parasite–host dynamics and their interaction with environmental conditions can contribute to the maintenance of healthy ecosystems in a time of rapid environmental change. A priority for monitoring programs is to investigate parasite transmission between wild and domestic species (Beck et al., 2017; Ciucci et al., 2020; Clark et al., 2018; Fuehrer et al., 2016; Otranto, Cantacessi, Dantas‐Torres, et al., 2015; Otranto, Cantacessi, Pfeffer, et al., 2015), particularly in response to environmental change (Acevedo‐Whitehouse & Duffus, 2009; Scheffers et al., 2016). Further research could help clarify the extent to which naturally transmitted parasites (i.e., from natural wolf prey) versus anthropogenically transmitted parasites (transmitted from feeding on or contact with domestic animals or human refuse) influence the health of wolves and other carnivores. Recent findings also highlight the importance of monitoring recolonizing wolf populations, as wolf‐specific parasites could increase in prevalence following the return of wolves (Lesniak et al., 2018). Parasites might thus contribute to re‐establishing ecosystems toward a more naturalized state in ways not typically observed or reported by humans. Notably, the link we found between higher dietary diversity and reduced prevalence of trophic transmission (i.e., indirect life cycle) of parasites in wolves provides a novel line of evidence by which biodiversity may maintain ecosystem health (Civitello et al., 2015). Moreover, the positive relationship between wolf density and the prevalence of parasites with direct transmission provides support for a regulatory role of parasites in ecosystems. Combined, these findings highlight the importance of multihost–parasite associations in the maintenance of ecosystem health and underscore the importance of conservation and restoration strategies that promote not only species but also a diverse suite of trophic interactions and host–parasite associations.

## CONFLICT OF INTEREST

No conflict of interest.

## AUTHOR CONTRIBUTION

**Astrid V. Stronen:** Conceptualization (equal); Data curation (equal); Formal analysis (supporting); Funding acquisition (equal); Project administration (equal); Resources (equal); Writing‐original draft (equal); Writing‐review & editing (equal). **Barbara Molnar:** Conceptualization (equal); Data curation (equal); Formal analysis (supporting); Funding acquisition (equal); Project administration (supporting); Resources (equal); Writing‐original draft (supporting); Writing‐review & editing (supporting). **Paolo Ciucci:** Writing‐review & editing (supporting). **Chris T. Darimont:** Funding acquisition (equal); Writing‐review & editing (supporting). **Lorenza Grottoli:** Writing‐review & editing (supporting). **Paul C. Paquet:** Funding acquisition (equal); Writing‐review & editing (supporting). **Tim Sallows:** Resources (equal); Writing‐review & editing (supporting). **Judit E. G. Smits:** Writing‐review & editing (supporting). **Heather M. Bryan:** Conceptualization (equal); Data curation (equal); Formal analysis (lead); Funding acquisition (equal); Project administration (equal); Resources (equal); Writing‐original draft (equal); Writing‐review & editing (equal).

## Supporting information

Appendix S1

## Data Availability

Parasite data are available in the Dryad Digital Repository: https://doi.org/10.5061/dryad.3r2280gg9
