# Supplementary material for: Cross‐continental comparison of parasite communities in a wide‐ranging carnivore suggests associations with prey diversity and host density
Source: Ecol Evol. 2021 Jul 13;11(15):10338–52. doi: 10.1002/ece3.7837 (PMC8328421; doi:10.1002/ece3.7837)
Supplement: Supplementary file 1 — Appendix S1 [file ECE3-11-10338-s001.docx]

**Appendix S1**

**Simulation of the potential effects of methodological differences on the detection probabilities of parasite eggs and oocysts in wolf faecal samples**

Our study drew on results derived using two different methods for the coprological investigation of parasite larval stages (i.e., eggs, oocysts, sporocysts, and nematode larvae). Samples from the Abruzzo, Lazio and Molise National Park (PNALM) in Italy, Mercantour National Park (MNP) in France, and the Northern Range of Yellowstone National Park (YNP) in the United States were analysed using sedimentation. By contrast, samples from Canada, including the central coast of British Columbia in a region known as the Great Bear Rainforest (GBR), Riding Mountain National Park (RMNP) and Duck Mountain Provincial Park and Forest (DMPPF) were analysed using faecal flotation (see Methods for more details). To examine the potential effects of methodological differences on our results, we first identified correction factors from published comparisons of these two methods (Table S1; Öge et al., 2017; Wolf et al., 2014). We then randomly dropped or added positive samples for specific parasite taxa to the study areas where we used flotation to make the results comparable to those obtained using sedimentation. Finally, we re-made figures and re-ran analyses for parasites with direct and indirect lifecycles using the adjusted dataset and compared the findings with our original results.

Overall, results using the adjusted dataset were like those obtained using the original dataset. In the adjusted dataset, the faecal prevalence of parasites with direct lifecycles did not change in DMPPF due to the low overall prevalence of direct parasites (2.5%) in that study area, decreased from 6.7% to 6.0% in RMNP, and decreased from 8.4% to 7.5% in the GBR (Fig S1, S2). Importantly, the overall pattern of higher faecal prevalence of parasites with direct lifecycles with higher wolf density remained after applying the correction factors (Fig S1, S2). Similarly, the faecal prevalence of parasites with indirect lifecycles changed only slightly after the correction factors were applied and the overall trend of lower faecal prevalence of parasites with indirect lifecycles with higher diet diversity remained (Fig S1B, S2B). Compared with the original dataset, the faecal prevalence of parasites with indirect lifecycles in the adjusted dataset decreased from 59.6% to 58.4% in GBR, decreased from 59.9% to 59.5% in RMNP, and increased from 59.0% to 60.6% in DMPPF (Fig S1B). The minimal changes in the prevalence of parasites with indirect transmission is due primarily to the higher efficiency of flotation in detecting *Sarcocystis* sporocysts being balanced by the lower efficiency in detecting Taeniid eggs (Fig S1B). Results of the mixed effects modelling of the adjusted data were also similar to those obtained using the original data. The magnitudes and confidence intervals of the coefficient estimates changed only slightly, and the highest-ranking models remained the same for parasites with direct and indirect lifecycles (Table S2).

Our results from this simulation suggest that methodological differences would not greatly influence the key findings of our paper. Nonetheless, the studies we used to determine correction factors were conducted on different hosts (reptiles [Wolf et al., 2014] and dogs [Öge et al., 2017], which mean results may have differed for wolf faeces. Moreover, we assumed that related parasite taxa would respond similarly to the two different methods, which is likely an over-simplification. Further, the methods described in the reference papers we used likely differed somewhat from the methods we applied, which would influence the correction factors. In the absence of direct comparison of methods on our own samples (which no longer exist), we believe this simulation provides added support of the variation in faecal prevalence of parasites that we observed among study areas.

Fig. S1. Percentage of wolf parasites with direct and indirect life cycles per study area in relation to estimated wolf density (individuals per 1000 km^2^) and dietary diversity **using data corrected for methodological differences (compare with Fig 2)**. Study areas are Abruzzo National Park (PNALM, Italy), Mercantour National Park (MNP, France), Yellowstone National Park (YNP, US), the Great Bear Rainforest (GBR) in coastal Canada, and Riding Mountain National Park (RMNP) and Duck Mountain Provincial Park and Forest (DMPPF) in continental Canada. Study areas are ordered by increasing wolf density (Panel A) and diet diversity (Panel B).


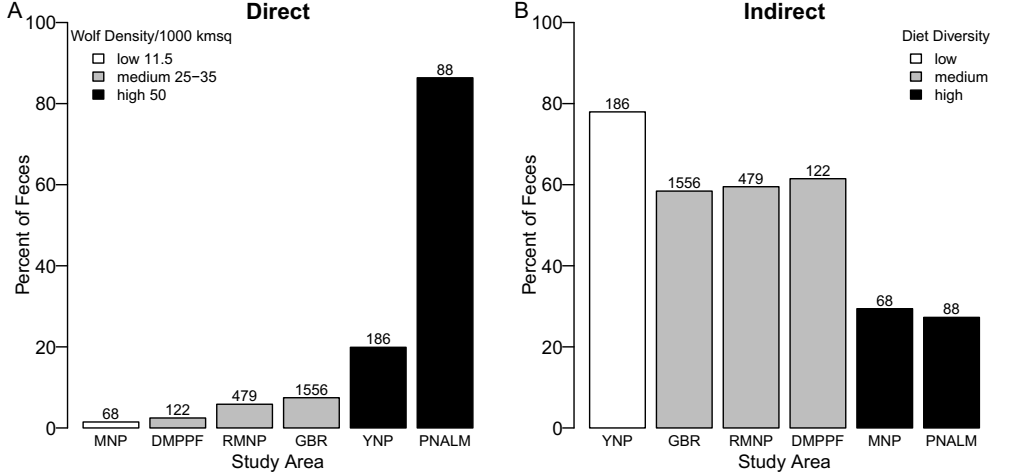


Fig. S2. Percentage of wolf parasites with direct (A) and indirect (B) lifecycles summarized for each study area **using data corrected for methodological differences (compare with Fig 3)**. Study areas include Abruzzo National Park (PNALM, Italy), Mercantour National Park (MNP, France), Yellowstone National Park (YNP, US), the Great Bear Rainforest (GBR) in coastal Canada, and Riding Mountain National Park (RMNP) and Duck Mountain Provincial Park and Forest (DMPPF) in continental Canada. Study areas are ordered by increasing wolf density (Panel A) and dietary diversity (Panel B). For panel A, ‘Other’ includes parasite taxa *Toxocara canis*, *Trichuris* spp., Spiroidea, and Coccidia. Not shown in panel B are parasite taxa *Metorchis* spp., which occurred in 0.6% of samples from the GBR and *Physaloptera* spp., which occurred in 2.3% of samples from PNALM.


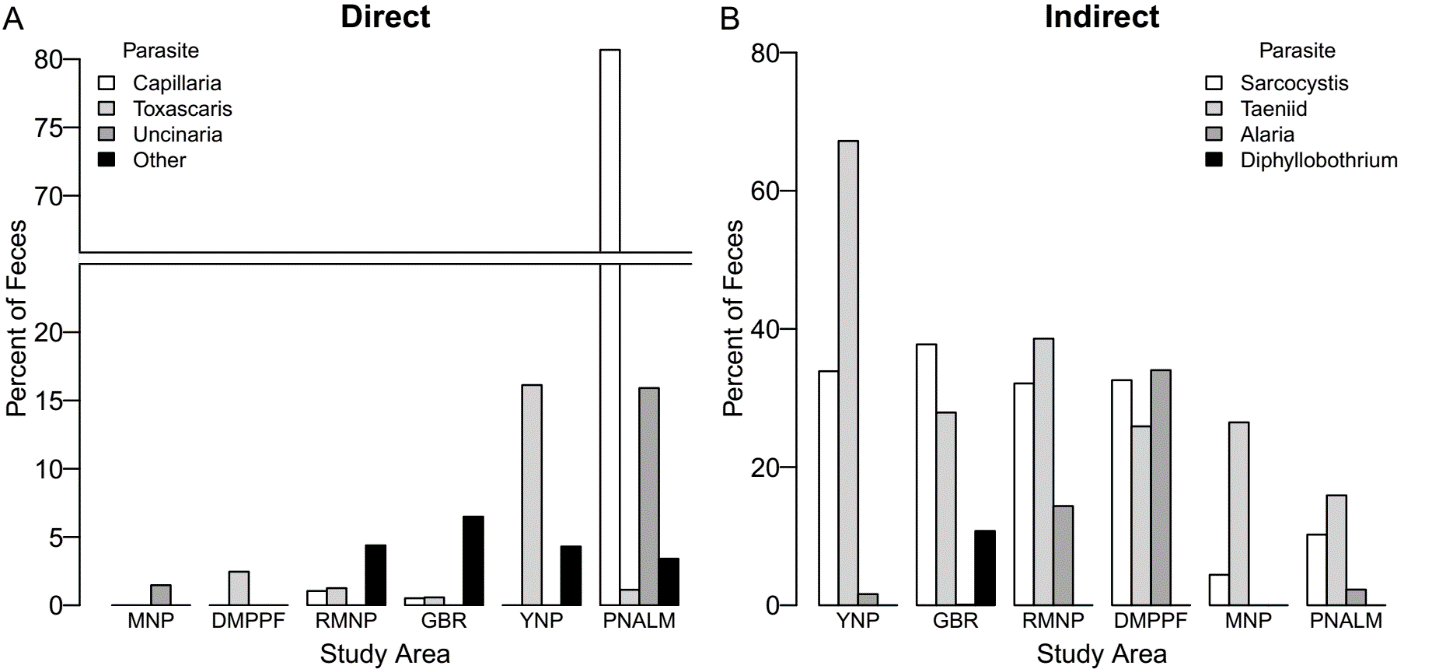


Table S1. Correction factors applied to the original dataset to simulate potential consequences of methodological differences between study areas. A positive correction factor indicates that samples positive for a particular parasite taxon were randomly removed whereas a negative correction factor indicates that positive samples were randomly added to the dataset. Correction factors were applied to study areas where faecal flotation was used for parasitological analyses, including GBR, DMPPF, and RMNP, unless otherwise noted.

| **Parasite Lifecycle** | **Taxon** | **Reference** | **Correction Factor** | **Notes** |
| --- | --- | --- | --- | --- |
| Direct | Nematode eggs (*Toxascaris leonina*, *Capillaria* spp., *Toxocara canis*, *Trichuris* spp., *Uncinaria* spp., and Spiroidea) | Wolf et al., 2014 | -13.6% | No correction applied to *Uncinaria* spp., as this taxon only detected in PNALM and MNP or to Spiroidea, as this taxon only detected in 2 samples from the GBR |
|  | Protozoan oocysts (Coccidea) | Wolf et al., 2014 | -13.6% | Applied to GBR only; Coccidian oocysts not detected elsewhere |
| Indirect | Protozoan sporocysts *(Sarcocystis* spp.) | Wolf et al., 2014 | -13.6% |  |
|  | Cestode eggs (Taeniids and *Diphyllobothrium* spp.) | Öge et al., 2017 | +17.0% | Correction for *Diphyllobothrium* only applied to GBR, as this taxon not detected elsewhere |
|  | Trematode eggs (*Alaria* spp. and *Metorchis* spp.) | Wolf et al., 2014 | +18.6% | Correction for *Alaria* spp. only applied to DMPPF and RMNP as this taxon not detected in the GBR; Correction for *Metorchis* spp. only applied to GBR |
|  | Nematode eggs *(Physaloptera* spp.) |  | None | Correction not applied, as this taxon only detected in PNALM |

Table S2. Results of models using data corrected for methodological differences in faecal parasitology among study areas for comparison with models using the original data in Table 4. Shown are candidate models for predicting the presence of parasites with direct or indirect lifecycles in wolf faeces, Akaike Information Criterion (AICc) ranking and weight, coefficient estimates and standard error (SE), and 95% Confidence Intervals (CI). Study area was included in all models as a random effect. Wolf density, dietary diversity, and number of alternative hosts were centred and scaled to have a mean of 0 and standard deviation of 1. History of recolonization was coded as “Recent” or “Not Recent”.

| **Response** | **Predictor** | **df** | **AICc** | **ΔAICc** | **Model**  **Weight** | **Estimate ± SE** | **Lower, Upper 95% CI** |
| --- | --- | --- | --- | --- | --- | --- | --- |
| Parasites with direct lifecycles | Wolf Density | 3 | 1361.68 | 0 | 0.88 | 1.98 ± 0.55 | 0.92, 3.05 |
|  | Null (~1) | 2 | 1367.41 | 5.73 | 0.05 |  |  |
|  | Diet Diversity | 3 | 1368.81 | 7.13 | 0.02 | 0.70 ± 0.85 | -0.97, 2.37 |
|  | History of Recolonization | 3 | 1369.07 | 7.72 | 0.02 | -1.02 ± 1.76 | -4.46, 2.43 |
|  |  |  |  |  |  |  |  |
|  | Alternative Hosts | 3 | 1369.40 | 7.69 | 0.02 | -0.13 ± 1.06 | -2.21, 1.95 |
|  |  |  |  |  |  |  |  |
| Parasites with indirect lifecycles | Diet Diversity | 3 | 3318.97 | 0 | 1 | -0.80 ± 0.13 | -1.07, -0.56 |
|  | Null (~1) | 2 | 3331.34 | 12.38 | 0 |  |  |
|  | Wolf Density | 3 | 3332.87 | 13.90 | 0 | 0.22 ± 0.34 | -0.46, 0.89 |
|  | Alternative Hosts | 3 | 3332.93 | 13.96 | 0 | 0.27 ± 0.39 | -0.51, 1.04 |
|  | History of Recolonization | 3 | 3333.31 | 14.34 | 0 | 0.14 ± 0.68 | -1.20, 1.47 |
